# An On-Chip Balun Using Planar Spiral Inductors Based on Glass Wafer-Level IPD Technology

**DOI:** 10.3390/mi16040443

**Published:** 2025-04-09

**Authors:** Jiang Qian, Peng Wu, Haiyang Quan, Wei Wang, Yong Wang, Shanshan Sun, Jingchao Xia

**Affiliations:** 1 School of Integrated Circuits, Peking University, Beijing 100871, China; qianj@mxtronics.com (J.Q.);; 2 Beijing Microelectronics Technology Institute, Beijing 100076, China

**Keywords:** on-chip balun, planar spiral inductors, glass, IPD

## Abstract

As integrated electronic microsystems advance, their internal components demonstrate increasing miniaturization, higher-density integration, and, consequently, significantly enhanced performance. This paper presents an on-chip transformer balun. The balun has a combination of planar coupled inductors and filtering capacitors using integrated passive device (IPD) technology, giving it the advantages of a more compact circuit size and lower cost to achieve single-ended to differential function on glass substrates. Moreover, it can be integrated in systems by flip-chip. The die has a size of 1.81 mm × 1.36 mm with a −15 dB single-ended return loss bandwidth of 2.07 GHz to 4.30 GHz. Within this bandwidth, the maximum insertion loss is 2.56 dB, and the amplitude imbalance is less than 2.04 dB. The phase difference between the differential signals is 180 ± 14.02° and the common mode rejection ratio (CMRR) is above 19.08 dB. The balun has the potential of miniaturization for integration on package or through-glass interposers (TGIs).

## 1. Introduction

Nowadays, with the development of More than Moore, integrated circuit packages tend to have high integration density, high performance, low power consumption, low size, and low cost [1]. In traditional integrated systems used by 2.5D integrated packaging technology, chips are usually integrated in a through-silicon interposer TSI [2]. However, it becomes increasingly difficult for TSIs to integrate different kinds of chips, especially in radio frequency (RF) microsystems, because of their cost and the limited electronics performance. In recent years, through-glass interposer (TGI) technology has been rapidly developed as a substitute for TSI due to its low substrate loss in the RF or microwave range, its mechanical robustness, and its low material and manufacturing cost [3]. In addition, glass is being increasingly utilized as a substrate material in the packaging of integrated circuit systems [4,5].

As more types of chips are integrated, the microsystems generate signal crosstalk and power interference that may affect their normal operation [6]. The performance of active devices in regards to linearity, noise figure, amplitude, and phase balance is usually not satisfactory [7]. Passive devices provide a solution to these problems by decoupling, filtering, matching the impedance, isolating power supply, and distributing power in the systems [8]. However, it is difficult for passive devices to achieve these functions or performance, which is determined by their size, as microsystems are becoming smaller [9,10,11]. With the development of wafer-level integrated passive device (IPD) technology, it is possible to integrate balun in smaller microsystems [12,13,14]. Cong demonstrated a compact 0.8 mm × 0.7 mm WLAN balun fabricated with integrated passive devices (IPDs) on a GaAs-on-Si substrate [15]. The balun uses a small outline transistor packaging technology to realize an excellent RF performance. Chang presented a compact transformer-coupled balun bandpass filter using silicon-based integrated passive device technology, which has the advantages of a more compact circuit size and lower cost [16]. Gao presented a balun device, consisting of a T-type low-pass filter and a π-type high-pass filter, fabricated through integrated passive technology on a GaAs substrate for S-band RF systems [7]. Due to IPD, a small size of 940 μm × 907 μm was achieved.

This paper reports an on-chip balun using planar spiral inductors based on glass wafer-level IPD technology which can be batch manufactured. The size is 1.81 mm × 1.36 mm, and the 10 dB single-end return loss (S11) bandwidth is 1.81 GHz~4.57 GHz. Within the bandwidth, the insertion loss (IL), amplitude difference, phase difference, and common mode rejection ratio (CMRR) are IL ≤ 2.56 dB, 2.04 dB, 14.02°, and 19.08 dB, respectively. The reported balun shows a miniaturization application potential in RF microsystems and other noise-sensitive microsystems.

## 2. Design and Simulation

**Figure 1:**
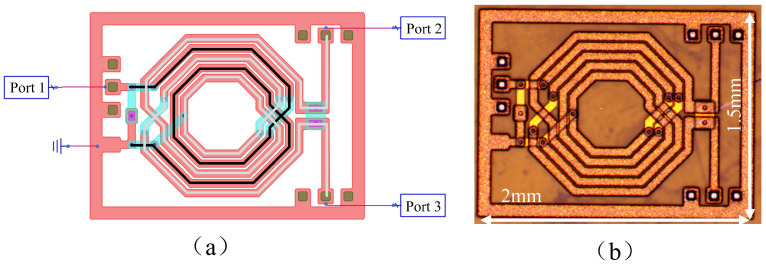
The proposed balun: (**a**) the schematic of the balun based on planar spiral coupled inductors and (**b**) the photograph of the balun.

The proposed balun consists of two coupled on-chip planar spiral inductors and three metal-insulator-metal (MIM) capacitors. One of the metal-insulator-metal (MIM) capacitors was placed on a balanced port and two capacitors were placed in unbalanced ports. Figure 1a shows the structure of the balun. The black line in Figure 1a represents the route of the primary inductor, while the gray line indicates the route of the secondary inductor. The secondary inductor is center-tapped, with its tap grounded. The cross-sectional structure of the balun is illustrated in Figure 1b. The balun was made in glass substrate and the dielectric layer material is polyimide (PI). There are two metal layers (M1, M2) used to construct the shape of the two coupled planar spiral inductors and provide the upper and lower plates of the MIM capacitors.

The balun in this article is a structure that converts single-ended signals to differential signals. The core part is equivalent to two transformers in principle, one connected in-phase and the other in anti-phase. In the case of ideal conductors and dielectric materials, when the coupling coefficient between the input port inductors and that at the output port is 1, the circuit insertion loss is 0 dB, indicating no energy loss during transmission. However, during actual manufacturing or design processes, this coupling coefficient is less than 1, leading to additional leakage inductance. Therefore, another approach to reduce energy loss is to enhance the quality factor of the coupling inductor. A higher quality factor not only minimizes energy dissipation but also improves impedance matching and bandwidth characteristics within the operating frequency range, thereby enhancing overall transmission efficiency and signal integrity.

**Figure 2:**
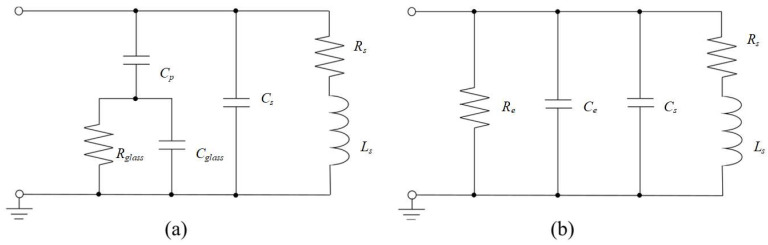
Lumped physical model of a spiral inductor: (**a**) original model and (**b**) equivalent model.

The lumped model of a single on-chip spiral inductor is depicted in Figure 2a. *L_s_* denotes the spiral inductance that can be calculated using the Greenhouse method [14]. *C_s_* refers to the capacitance resulting from the overlaps between the spiral and the center-tap underpass. *C_p_* stands for the PI capacitance between the spiral inductor and the substrate. *C_glass_* and *R_glass_* denote the capacitance and resistance of the glass substrate, respectively. Figure 2b illustrates the equivalent circuit of Figure 2a, where *C_p_*, *C_glass_,* and *R_glass_* are modeled as *R_e_* and *C_e_*, which exhibit frequency-dependent characteristics.

In Figure 1b, according to the definition of the quality factor for an inductor, the quality factor *Q* can be expressed as [17]

(1) $$Q=2\cdot \pi \cdot \frac{E_{pm}-E_{pe}}{E_{loss}}=\frac{\omega \cdot L_{S}}{R_{S}}\cdot \frac{R_{e}}{R_{e}+[{\left(\frac{\omega \cdot L_{S}}{R_{S}}\right)}^{2}+1]}\cdot [1-\frac{R_{S}^{2}\cdot (C_{S}+C_{e})}{L_{S}}-\omega^{2}\cdot L_{S}\cdot (C_{S}+C_{e})]$$

where

(2) $$R_{e}=\frac{1}{\omega^{2}C_{P}^{2}R_{glass}}+\frac{R_{glass}\cdot {\left(C_{P}+C_{glass}\right)}^{2}}{C_{glass}^{2}}$$

(3) $$C_{e}=C_{P}\cdot \frac{1+\omega^{2}\cdot (C_{P}+C_{glass})\cdot C_{glass}\cdot R_{glass}^{2}}{1+\omega^{2}\cdot {\left(C_{P}+C_{glass}\right)}^{2}R_{glass}^{2}}$$

and where *R_s_* represents the metal series resistance influenced by eddy current effects, and is given by [15]

(4) $$R_{s}(\omega )=\frac{l}{\omega \cdot \sigma \cdot \delta (\omega )\cdot (1-e^{\frac{t}{\delta }})}$$

(5) $$\delta (\omega )=\sqrt{\frac{1}{\omega \cdot \mu \cdot \sigma }}$$

and where *μ* and *σ* are permeability and electrical conductivity of the inductor material, respectively. *δ* is the skin depth of the inductor. *E_pm_* = *V*_0_^2^·L_s_/{2·[(*ω*·*L_s_*)^2^ + *R_s_*^2^]} is the peak magnetic energy in the inductor, *E_pe_* = *V*_0_^2^·(*C_s_* + *C_e_*)/2 is the peak electric energy. According to Equation (1), the second term is the substrate loss factor, and the last term denotes the inductive self-resonance factor. Therefore, reducing *R_e_* can decrease the energy dissipated in the substrate.

**Figure 3:**
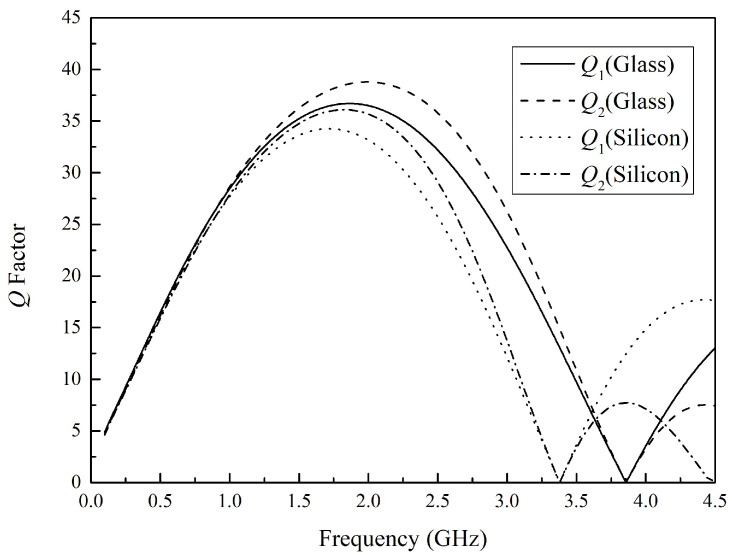
Quality factor simulation analysis of glass-based and silicon-based inductors.

The primary inductance and secondary inductance in Figure 1 are modeled in a three-dimensional electromagnetic field, with the substrate materials being silicon and glass, respectively. The width of the inductors is 50 μm, and the line pitch is 15 μm. The simulation results of the *Q* factor are shown in Figure 3. The frequency was set from 100 MHz to 4.5 GHz, which covers the basic requirements for the frequency range of wireless communication systems and RF systems. *Q*_1_ and *Q*_2_ represent the primary inductor and second inductor, respectively. According to Figure 3, the *Q* factor of glass-based inductors is almost the same as that of silicon-based inductors below 1.5 GHz. In addition, during the transition from low to high frequencies, inductors shift from exhibiting inductive to capacitive characteristics. Despite this capacitive behavior, mutual coupling persists between inductors, enabling the continuous realization of single-ended to differential signal conversion functionality. However, the disadvantage of silicon-based inductors will only be reflected when the frequency increases. Moreover, the working frequency range of the balun in this article is more suitable for using glass-based inductors. Therefore, the result can be attributed to the lower magnetic permeability, higher resistivity, and reduced surface roughness of glass substrates, which collectively minimize energy losses and enhance magnetic field retention.

**Figure 4:**
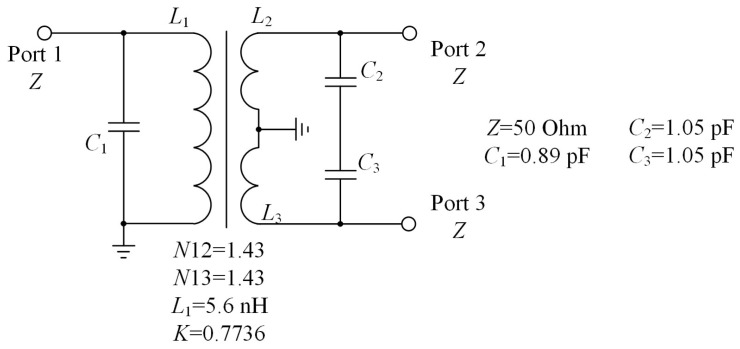
The circuit diagram which is the design goal of the reported balun.

**Figure 5:**
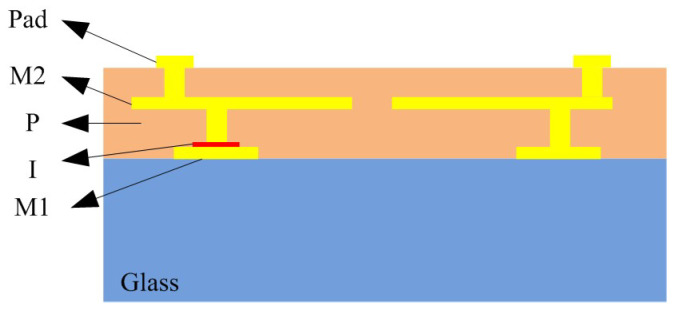
The cross-side view of the balun based on glass substrate. The yellow part represents metal materials, the orange part represents dielectric materials, and the blue part represents glass materials.

**Figure 6:**
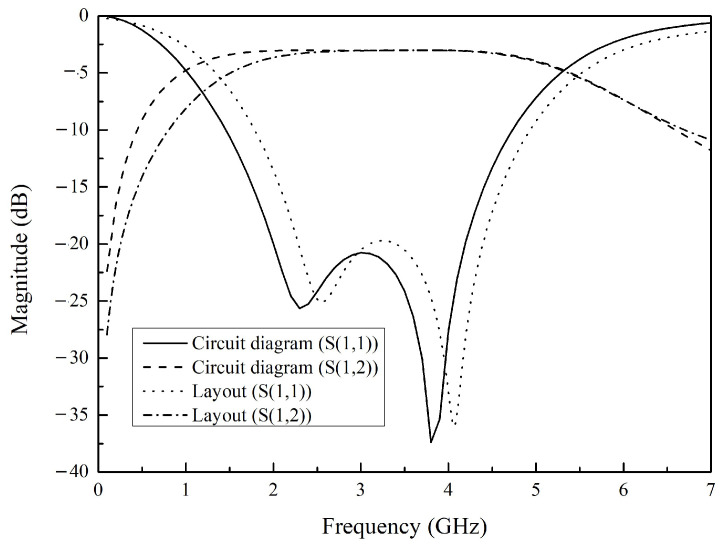
Model of the balun for S-parameter simulation results.

We combined planar spiral coupled inductors and capacitors to design a balun as shown in Figure 4. The figure shows the turns ratio of the coupled inductor, the inductance value of the primary coil, the capacitance values of the balanced port, and the capacitance value of the unbalanced port, respectively. The frequency range of the balun is set from 2 GHz to 4GHz, which covers most ap-plication scenarios of RF microsystems, in order to ensure the function of impedance matching. Figure 5 shows the stack layers of the balun used in simulation of circuit diagram. In order to preserve the design margin considering manufacturing errors and ensure impedance matching performance, we paid attention to the bandwidth corresponding to −15 dB during the design. The simulation results are shown in Figure 6. As can be seen from the figure, within the frequency range of 1.77 GHz to 4.39 GHz, S11 is consistently below −15 dB, and S12 is always above −2.50 dB.

The MIM capacitors consisted of an M1 layer, I layer, metal via, and M2 layer. The coupling inductors were created in the M1 layer, metal via, and M2 layer. According to Figure 1a, the balanced port is set to Port 1, and the unbalanced port is configured as Port 2. The outer side is a ring ground in layer M2. The values used in the layout simulation of the layers’ parameters are shown in Table 1. In addition, the conductivity of copper is provided by [18]. The relative permittivity, dielectric loss tangent, and conductivity of the polymide, SiN_x_, and glass are provided by process testing from the manufacturer.

The transmission performance of the layout shown in Figure 1 was simulated, and the results are shown in Figure 6. As can be seen from the figure, within the frequency range of 2.07 GHz to 4.60 GHz, S11 is consistently below −15 dB, and S12 is always above −3.26 dB. The deviation between the simulation results of the layout and the circuit diagram is caused by additional parasitic effects on the layout.

## 3. Processing

**Figure 7:**
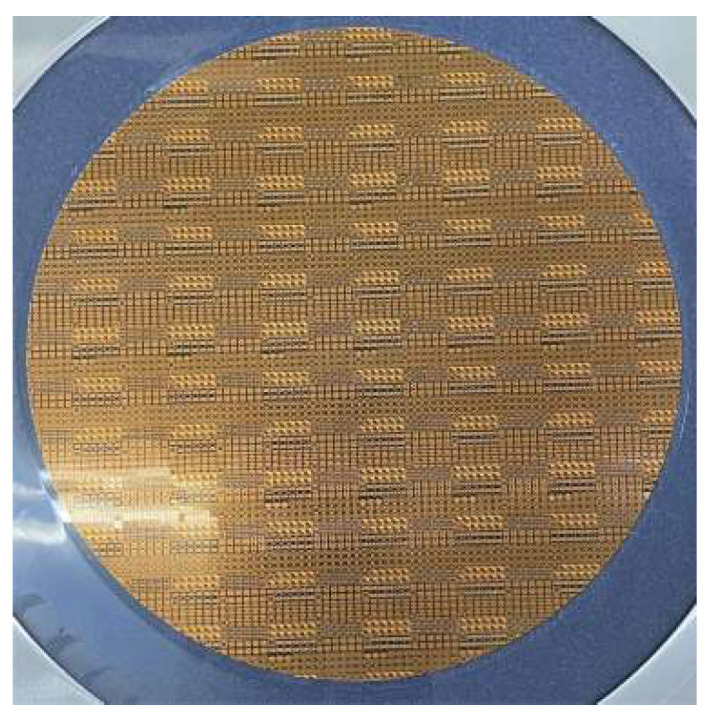
Photograph of the balun chips (8-inch glass wafer).

The glass substrate-based wafer-level IPD technology presented in this paper utilizes a 12-inch glass wafer as the base material, which can significantly reduce both capacitive and inductive loading caused by conductive substrates in high-speed microelectronic applications. The manufacturing process begins with depositing metallic Cu at a thickness of 5 μm on the glass wafer using physical vapor deposition (PVD). Next, the wafer is subjected to photolithography utilizing photoresist materials for masking in order to define and form the bottom metal structure, which primarily includes a small portion of the secondary inductor routing and the lower plate of the MIM capacitor. The portion allocated for the secondary inductor coil wiring is specifically designed for interlayer switching during the crossing of the secondary inductor and the primary inductor coil traces. Thirdly, a 0.2 μm-thick silicon nitride (SiN_x_) layer is deposited as the intermediate dielectric layer of the MIM capacitor, followed by photolithographic masking to pattern the structure. Next, a polyimide (PI) layer with a thickness of 20 μm is deposited, exposing the central areas of both the underlying metal and the MIM intermediate dielectric layer. Finally, after depositing a seed layer followed by electroplating copper (Cu), the topmost structure is constructed, which includes the upper plate of the MIM capacitor, the primary inductor coil, the remaining portion of the secondary inductor coil, and interconnect metal routing. Figure 7 shows the fabricated balun chips, which demonstrates the advantage of batch fabrication. It can be seen from Figure 7 that the balun designed in this article has advantages of high consistency and batch production by wafer-level IPD technology.

## 4. Results

**Figure 8:**
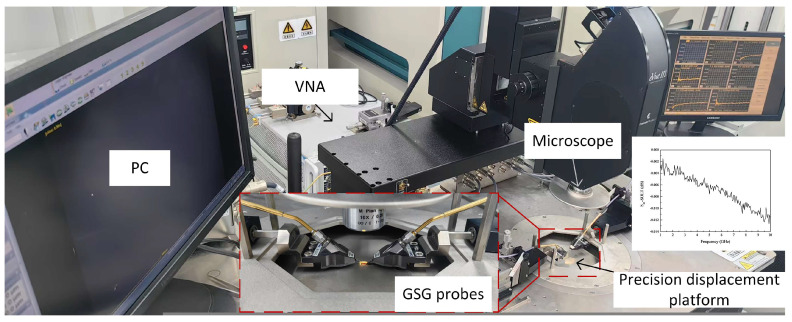
The test system of the balun: PC (used to control the focus and displacement of the microscope), microscope (visual positioning), G-S-G probe (used to provide signal transmission and 50-ohm impedance matching), and VNA (used to measure S-parameter).

The fabricated balun was subjected to performance testing, with a focus on evaluating its frequency response and balance characteristics. We tested the balun using the system shown in Figure 8, consisting of ground-signal-ground (G-S-G) probes, vector network analyzers (VNAs), a microscope, and display devices. The measurements were conducted on a Cascade Summit 12000 probe station (Cascade Microtech, Beaverton, OR, USA) with a ground-signal-ground (GSG) probe (Ceyear GSG-150-μm-pitch-40-GHz, Ceyear Technologies, Qingdao, China) and a Keysight 5247A vector network analyzer (Keysight Technologies, Calabasas, CA, USA). Prior to testing, a short-open-load-through (SOLT) calibration was performed on the probes using a GGB calibration substrate (model: GSG-CS-5, GGB Industries, Naples, FL, USA) to ensure measurement accuracy. Post-calibration, the reference plane was aligned at the probe tip. Following the SOLT calibration, the thru standard was characterized to validate calibration integrity. As is shown in Figure 8, the measured S21 magnitude exhibited a maximum deviation of less than 0.013 dB across the 1–10 GHz frequency range, which meets the accuracy requirements for the experimental characterization in this work.

The test frequency range was set from 100 MHz to 10 GHz, and the S-parameters, amplitude imbalance, and phase imbalance of both the balanced and unbalanced ports were measured. The amplitude imbalance and phase imbalance measurement data can be calculated by [19]

(6) $$Amplitude\;imbalance=20\cdot \log_{10}\left|\frac{S_{31}}{S_{21}}\right|$$

(7) $$Phase\;imbalalce=ang\left(\frac{S_{31}}{S_{21}}\right)$$

where *S*_21_ and *S*_31_ are the measurement values. The results are presented in Figure 7, which also includes simulated values for comparison purposes.

**Figure 9:**
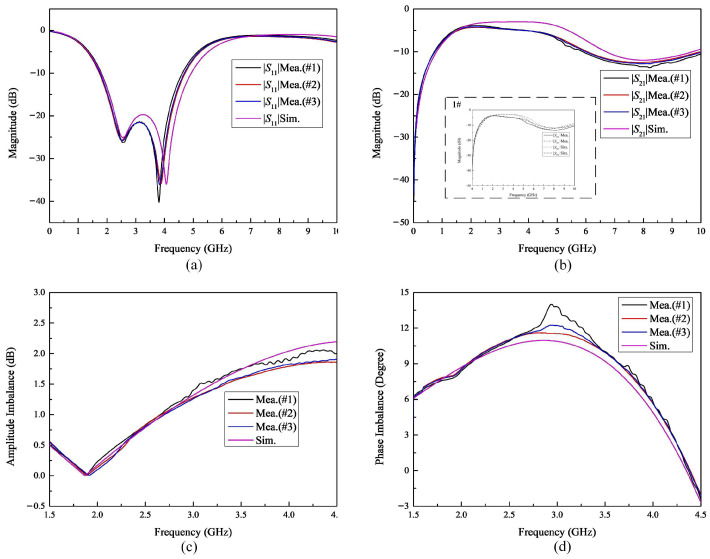
The performance of the balun on glass: (**a**) insertion loss, (**b**) return loss, (**c**) magnitude imbalance, and (**d**) phase imbalance.

We measured three fabricated baluns. Balun #1 was positioned at the outermost edge of the wafer, while Balun #3 was placed at the wafer center. Balun #2 was sampled from a region between the center and edge of the wafer. The results are shown in Figure 9. The repeatability/variation of the manufacture can be observed by the results. The following discussions on performance are all focused on Balun #1. Figure 9a illustrates the relationship of the frequency and the return loss (S11), revealing that, within the frequency range of 2.07 GHz to 4.30 GHz, S11 remains below −15 dB, with a minimum value of approximately −40 dB. Moreover, as depicted in Figure 9b, the maximum insertion loss is 2.56 dB at 4.30 GHz within the corresponding frequency range. In fact, according to Figure 1b, due to the larger line width, the roughness of the conductor edge caused by photolithography increases high-frequency resistance, resulting in significant insertion loss. During simulation, the layout shows that the edges of the conductors are smooth. Figure 9c and Figure 9d present the measurement results of the amplitude imbalance and phase imbalance, respectively. The amplitude imbalance reaches a maximum value of 2.05 dB at 4.30 GHz. Meanwhile, the phase imbalance reaches a maximum value of 14.02° at 2.94 GHz.

The linearity and electromagnetic compatibility (EMC) of the balun transformer were evaluated using the common-mode rejection ratio (*CMRR*), and the results are shown in Figure 8. The *CMRR* is given by [20]

(8) $$CMRR=\frac{DMG}{CMG}=\frac{(S_{21}-S_{31})/\sqrt{2}}{(S_{21}+S_{31})/\sqrt{2}}$$

**Figure 10:**
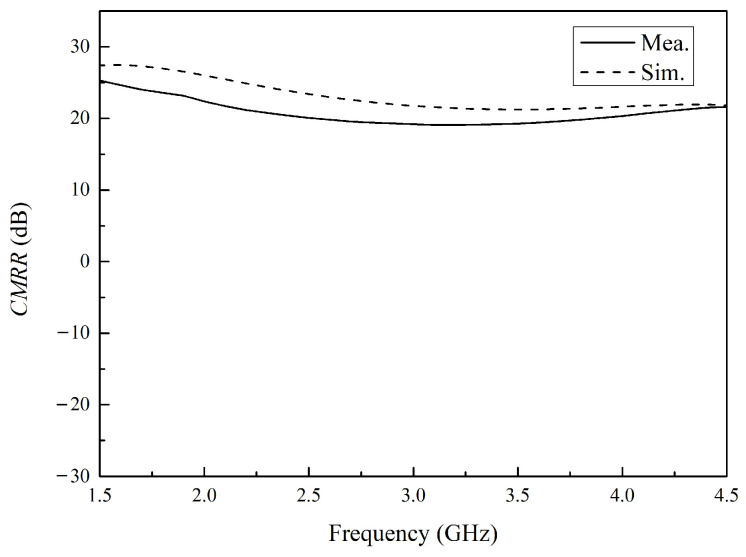
The *CMRR* (Common-Mode Rejection Ratio) of the balun. The solid line denotes the measured results of the balun, while the dashed line corresponds to the simulated results from its layout model.

As is shown in Figure 10, the dashed line represents the simulated *CMRR* values, while the solid line denotes the experimentally measured data. The measured *CMRR* values exhibit a maximum of 19.08 dB, whereas the simulated values are above 21.82 dB. The discrepancy between the simulated and measured results are attributed to the influence of parasitic components in the actual implementation. The simulation results and measurement results of *CMRR* exhibit identical trends. The proposed design demonstrates promising *CMRR* performance, indicating its potential for applications requiring high common-mode rejection.

## 5. Discussion

The amplitude imbalance in Figure 9c is high. This is because the magnetic field gradient enhancement and proximity effect of the coils increase eddy current losses due to the small pitch of the coil (15 μm). The phase imbalance in Figure 9d is attributed to the asymmetrical structure of the secondary coils caused by the crossover routing at the intersection of the primary and secondary coils on the same layer. Therefore, increasing the spacing and symmetrical coil layer transition routing can optimize the amplitude imbalance and phase imbalance. The simulation results also confirm the phenomenon. Furthermore, as can be observed from Figure 9, the layout simulation results are generally in agreement with the measured results for the aforementioned parameters, demonstrating that the material and process-induced errors in the balun are relatively small. With IPD technology, the balun has the advantages of a more compact circuit size and lower cost to achieve single-ended to differential function on glass substrates. Furthermore, it can be integrated into System-in-Package (SiP) interposers using flip-chip technology. Moreover, the balun has the potential of miniaturization for integration on package or TGIs.

The reported transformer balun based on IPD technology is compared with those utilizing alternative technologies through the data presented in Table 2. As evidenced by the table, our proposed balun exhibits superior performance characteristics, particularly in terms of compact size and a high *CMRR*.

## 6. Conclusions

This paper reported an on-chip glass-based transformer balun based on spiral coupled inductors, fabricated by glass-based wafer-level integrated passive device (IPD) technology. The analysis of the on-chip coupled inductors revealed that glass-based inductors exhibit a relatively high *Q* factor. The experimental results demonstrated that the on-chip balun based on glass wafer IPD technology possesses advantages in terms of smaller size and higher CMRR compared to PCB techniques. These findings suggest significant potential for miniaturization application in RF microsystems and other noise-sensitive microsystems.

## Figures and Tables

**Table 1:** The Information of the layers in Figure 5.

Layer	Material	Thickness (μm)	Relative Permittivity	Dielectric Loss Tangent	Conductivity (S/m) [18]
Pad	Cu	8	/	/	5.80 × 10^7^
M2	Cu	5	/	/	5.80 × 10^7^
P	Polymide	20	3.16 (5 GHz)	0.0109 (5 GHz)	0
I	SiN_x_	0.2	6.8 (5 GHz)	0.0025 (5 GHz)	0
M1	Cu	5	/	/	5.80 × 10^7^
Glass	Glass	250	4.4 (5 GHz)	0.0057 (5 GHz)	0

**Table 2:** Comparison of measured specifications and size between the designed balun and other works.

	Technology	Bandwidth/GHz	Insertion Loss/dB	Amplitude Imbalance/dB	Phase Imbalance/(°)	Size (mm × mm)	CMRR /dB
[21]	PCB	1.19–3.25	<5.1	<0.49	<6.5	18 × 17.5	/
[22]	PCB	2–4 (>11 dB)	<0.75	<0.6	<7	0.85λ_g_^2^	>15
[23]	LTCC	0.8–3	<6	<0.7	<6	3.05 × 1.3	/
[24]	Multilayer configuration	0.5–1.3	1.0	/	180 ± 3%	3.2 × 1.6	/
[25]	LTCC	0.11–0.14	3.3	0.18	<1	8.5 × 9.5	**/**
This work	IPD	2.07–4.30	<2.50	<2	<14.02	1.81 × 1.36	>19.08

## Data Availability

The original contributions presented in the study are included in the article, further inquiries can be directed to the corresponding author.
